# ChroPac-Trial: Duodenum-preserving pancreatic head resection versus pancreatoduodenectomy for chronic pancreatitis. Trial protocol of a randomised controlled multicentre trial

**DOI:** 10.1186/1745-6215-11-47

**Published:** 2010-04-29

**Authors:** Markus K Diener, Thomas Bruckner, Pietro Contin, Christopher Halloran, Matthias Glanemann, Hans Jürgen Schlitt, Joachim Mössner, Meinhard Kieser, Jens Werner, Markus W Büchler, Christoph M Seiler

**Affiliations:** 1 University of Heidelberg, Department of General, Visceral and Transplantation Surgery, Heidelberg, Germany; 2 University of Heidelberg, Study Centre of the German Surgical Society, Heidelberg, Germany; 3 University of Heidelberg, Institute of Medical Biometry and Informatics, Heidelberg, Germany; 4 University of Liverpool, Divison of Surgery and Oncology, School of Cancer Studies, Liverpool, UK; 5 Charité University Hospital, Campus Virchow-Clinic, Department of General, Visceral and Transplantation Surgery, Germany; 6 University Medical Center Regensburg, Department of Surgery, Regensburg, Germany; 7 University of Leipzig, Medical Clinic and Polyclinic, Leipzig, Germany

## Abstract

**Background:**

A recently published systematic review indicated superiority of duodenum-preserving techniques when compared with pancreatoduodenectomy, for the treatment of patients with chronic pancreatitis in the head of the gland. A multicentre randomised trial to confirm these results is needed.

**Methods/Design:**

ChroPac aims to investigate differences in quality of life, mortality and morbidity during 24 months after surgery (duodenum-preserving pancreatic head resection versus pancreatoduodenectomy) in patients with chronic pancreatitis of the pancreatic head.

ChroPac is a randomised, controlled, observer and patient blinded multicentre surgical trial with two parallel comparison groups. The primary outcome measure will be the average quality of life during 24 months after surgery. Statistical analysis is based on the intention-to-treat population. Analysis of covariance will be applied for the intervention group comparison adjusting for age, centre and quality of life before surgery. Level of significance is set at 5% (two-sided) and sample size (n = 100 per group) is determined to assure a power of 90%.

**Discussion:**

The ChroPac trial will explore important outcomes from different perspectives (e.g. surgeon, patient, health care system). Its pragmatic approach promises high external validity allowing a comprehensive evaluation of the surgical strategy for treatment of patients with chronic pancreatitis.

**Trial registration:**

Controlled-trials.com ISRCTN38973832

## Background

### Rationale of the trial

**Figure 1:**
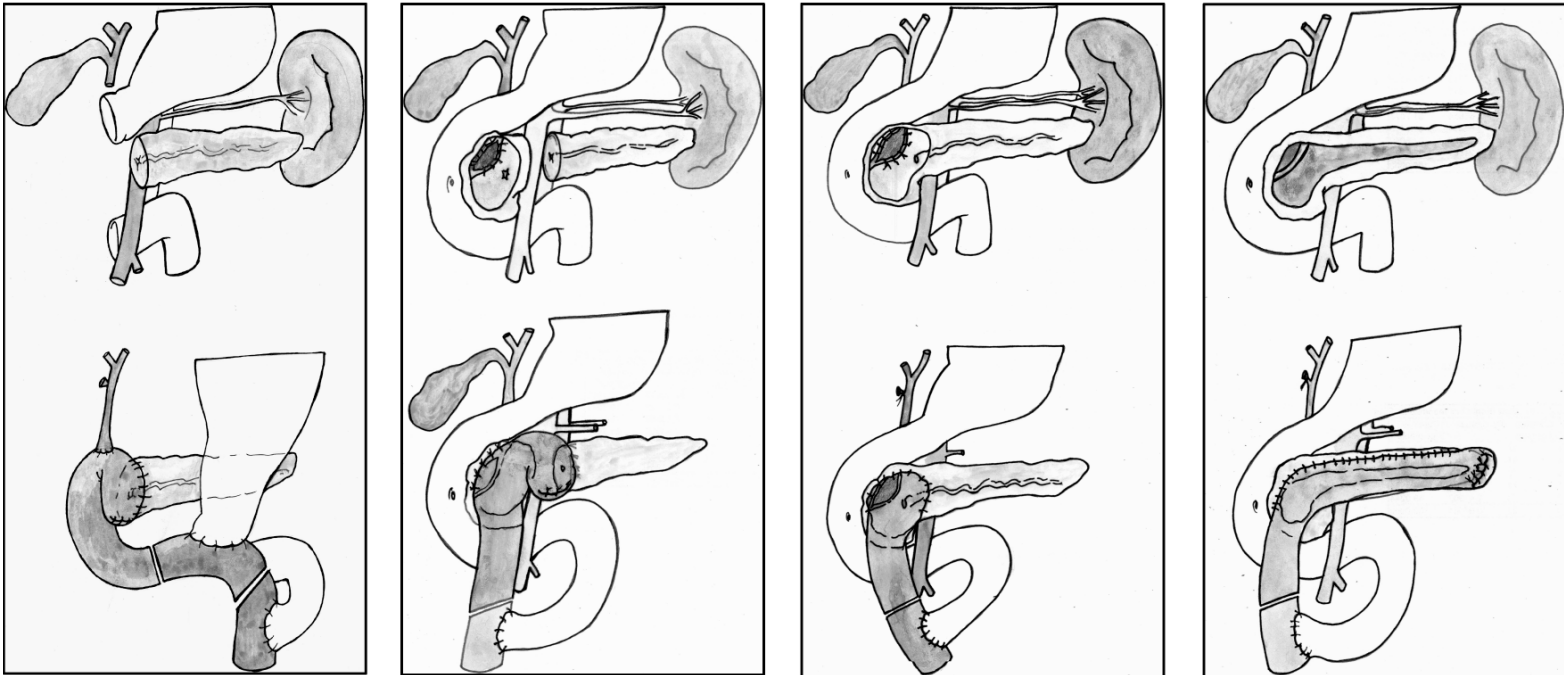
**Techniques of pancreatoduodenectomy and duodenum-preserving pancreatic head resection (DPPHR)**. (A) Classical Whipple procedure after resection and reconstruction with pancreaticojejunostomy, bilio-digestive anastomosis and gastrojejunostomy. (B) DPPHR according to Beger: Dissection of the pancreas above the portal vein, escavation of the pancreatic head and incision of the common bile duct. Reconstruction s accomplished by two pancreaticojejunostomies (corpus and pancreatic head). (C) DPPHR, Berne modification: Excavation of the pancreatic head and incision of the common bile duct without dissection of the pancreas above the portal vein. Reconstruction is accomplished by one single side-to-side pancreatcojejunostomy. (D) DPPHR, Frey modification: circumscripted excavation of the pancreatic head and longitudinal incision of the pancreatic duct. Reconstruction is accomplished by pancreaticojejunostomy.

Chronic pancreatitis (CP) is defined as a continuous inflammatory process causing permanent structural damage ultimately resulting in impairment of the gland's exocrine and endocrine function [1,2]. The most important causative agent is alcohol. Patients with CP who present with inflammatory pancreatic head enlargement, commonly require pancreatic head resection due to development of local complications (e. g. stenosis of the common bile duct and/or main pancreatic duct, duodenal obstruction, compression of retropancreatic vessels), suspicion of malignancy, and most commonly intractable pain [3,4]. Pancreatoduodenectomy (PD), i.e. the classical Whipple (CW) and subsequently the pylorus-preserving Whipple (PPW) procedure has served as primary surgical procedures for removal of the pancreatic head in patients with CP and pancreatic head enlargement for many years. However, PD is reported to have unsatisfactory outcome in terms of late morbidity; including high incidence of postoperative diabetes mellitus of up to 48%, attributed to the extensive resection of the duodenum and a larger portion of the pancreas [5]. In order to preserve the duodenum and limit resection of the pancreatic tissue to a minimum, the duodenum-preserving pancreatic head resection (DPPHR) was introduced by H. G. Beger in 1972 [6,7]. While the Beger procedure selectively removes the pancreatic head, two modifications of this procedure were developed in order to prevent dissection of the pancreas above the portal and superior mesenteric vein, a potential source of haemorrhage, particularly in case of portal hypertension: The Frey procedure consists of a local resection of the pancreatic head, which is combined with lateral pancreaticojejunostomy; in the Berne procedure the pancreatic head is resected subtotally, leaving a narrow layer of pancreatic tissue towards the duodenum and the retropancreatic vessels [4,8,9] (Figure 1).

### Preliminary data

A recently published randomised trial demonstrated superiority of surgical treatment (i. e. pancreaticojejunostomy) as compared with endoscopic drainage in patients with chronic pancreatitis and a distal pancreatic duct obstruction without an inflammatory mass, respectively [10]. Moreover, an up-to-date systematic review with meta-analysis indicated superiority of duodenum-preserving pancreatic head resection (DPPHR) compared with pancreatoduodenectomy (PD) in terms of several intra- (blood replacement) and postoperative (exocrine insufficiency, length of hospital stay, weight gain and occupational rehabilitation) outcome parameters as well as quality of life (QoL) [11]. However, this potential superiority of DPPHR is currently based on small hypothesis driven clinical trials performed in a single-institution setting and may be distorted by as well random error due to small sample sizes, as systematic sources of bias such as allocation concealment, standardization of study interventions, definition of outcome parameters, consistency of follow-up, and blinded outcome assessment. Large multicenter randomised trials comparing the two main strategies (DPPHR vs. PD) focussing on both the surgeons and patients perspectives are still missing.

### Objective

ChroPac aims to investigate differences in QoL during 24 months after surgery of DPPHR versus PD and mortality as well as general and pancreas-associated postoperative morbidity.

### Trial locations

ChroPac will be performed in thirteen trial sites with expertise for pancreatic surgery. Most of the participants were involved in a former multicentre-trial surgical trial for pancreatic left resection (DISPACT Trial, ISRCTN 18452029) carried out by the Study Centre of the German Surgical Society (SDGC). Thus the ChroPac trial group consists of international pancreatic surgeons at high-volume centres who also have expertise in trial conduction.

## Methods/Design

### Trial population and eligibility criteria

All patients scheduled for primary elective surgery for chronic head-pancreatitis will be eligible, assuming their ability to understand the character and individual consequences of participation as well as written informed consent. Patients participating in another interventional trial with potential interference of intervention (e.g. trials evaluating alternative perioperative analgetic regimens, application of octreotide etc.) and outcome (e.g. trials focussing on QoL) will be excluded.

### Sample size

A total of 200 patients will be randomised and included in the analysis.

### Type of Trial

Randomised, controlled, observer and patient blinded multicentre surgical trial with two parallel comparison groups.

### Recruitment and trial timeline

**Figure 2:**
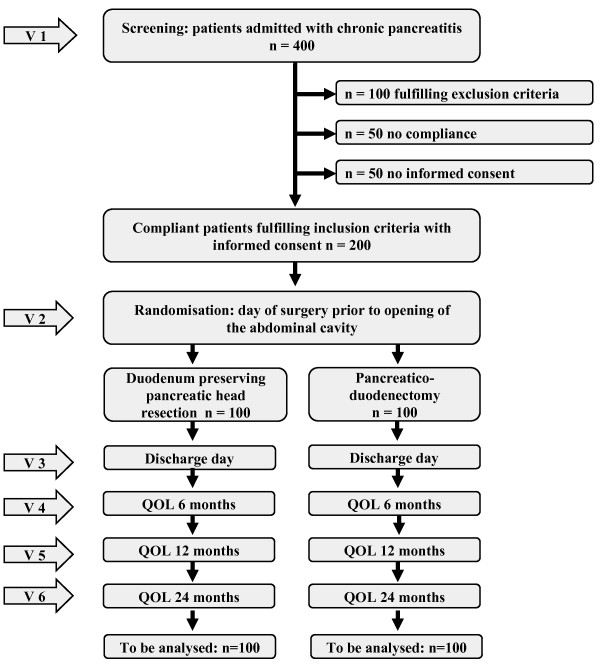
**ChroPac study flow**.

13 centres with high-level expertise for pancreatic surgery will contribute to recruitment of study patients. The duration of the trial for each participant is expected to be about 2 years. The duration of the overall trial is expected to be 5 years, including prearrangement and analysis. Recruitment of participants started in April 2009. The process of the trial conduct is illustrated in Figure 2.

### Randomisation

In order to achieve comparable intervention-groups, patients will be allocated concealed by preoperative randomisation at the day of surgery using a centralised web based tool http://www.randomizer.at. Block randomisation will be performed for each centre to achieve equal group sizes per centre.

### Blinding

Patients and outcome assessors will be blinded for the trial intervention.

### Interventions

#### Trial-intervention

Any surgical technique that removes inflamed pancreatic tissue of the head without resection of the duodenum (e.g. Begers, Frey or Berne procedure).

#### Control-Intervention

PPW or CW.

The procedures in both groups can be chosen according to preference of the surgeon which is based on his/her personal knowledge, skills and mainly the anatomical situation of the patient, since ChroPac aims to compare general surgical strategies rather than to compare certain modifications of the surgical procedure. Each participating centre performs the procedure according to local standards and according to the anatomical situation.

The schedule of trial interventions is presented in Table 1.

**Table 1:** Study visits of the ChroPac-Trial

Visit	Visit 1 (Screening)	Visit 2 (Day of surgery)	Visit 3 (Day of discharge)	Visit 4 (6 months post OP)	Visit 5 (12 months post OP)	Visit 6 (24 months post OP)
Demographics and baseline clinical data	X					

Inclusion/exclusion	X					

Randomisation		X				

Surgical intervention		X				

Assessment of secondary outcome measures and safety		X	X	X	X	X

Quality of Life	X			X	X	X

Tissue and blood sampling		X				

### Risks

No additional risks for study patients are awaited, since all surgical procedures carried out within ChroPac represent clinically established standard methods of treatment of chronic pancreatitis.

### Outcomes

The primary outcome measure of this trial is the average QoL during 24 months after surgery, measured 6, 12 and 24 months after surgery by the EORTC QLQ-C30 scale „physical functioning“ (PF-2). Secondary outcomes of: mortality, general (wound infection, pulmonary infection) and pancreas-associated postoperative morbidity (pancreatic fistula, delayed gastric emptying), operation time, intraoperative blood loss, initial postoperative hospital stay, reoperation, hospital stay related due to chronic pancreatitis within 24 months after randomisation, weight gain, development of exocrine insufficiency and new onset of diabetes mellitus will be assessed (Table 2). All outcome variables will be evaluated according to internationally accepted standards and scoring systems if available, i.e. the consensus definitions for pancreatic fistula and delayed gastric emptying of the International Study Group of Pancreatic Surgery (ISGPS) [12,13].

**Table 2:** Summary and definitions of secondary outcomes

Summary and definitions secondary outcomes	
**Mortality**	Death due to any cause at any time during the follow-up period including reason.

**General morbidity**	
▪ Wound infection	Surgical site infections will be assessed at discharge and 6 months after surgery, divided into superficial and deep incisional surgical site infection according to the Center for Disease Control and Prevention definition [22].
▪ Pulmonary infection	Post-Op-Pulmonary infection will be assessed, at discharge and 6 months and is defined according to local standards:
	Infection of the lung with either evidence of increased infection parameters (CRP > 2 mg/dl and/or leukocytes > 10 0000/ml) which are not caused by a different pathologic process or evidence of pulmonary infiltration in the chest x-ray, requiring antibiotic therapy.

**Pancreas associated postoperative morbidity**	
▪ Postoperative Pancreatic fistula [12]	Any abnormal communication between the pancreatic ductal epithelium and another epithelial surface containing pancreas-derived, enzyme-rich fluid.
	It should satisfy the following criteria:
	• output through an operatively placed drain or a subsequently placed percutaneous drain of any measurable volume of drain fluid
	• on or after postoperative day 3
	• amylase content in fluid greater than three times the upper normal serum value.
	Since only longstanding observation will confirm the diagnosis, it is necessary to distinguish and to grade the POPF as grades A, B and C after clinical recovery is complete.
	Grade A:
	• Without clinical impact
	• Oral nutrition
	• No antibiotics
	• No somatostatin analogues
	• No peripancreatic fluid collection
	• No delay in hospital discharge
	Grade B:
	• Clinically relevant
	• Partial/total parenteral/enteral nutrition
	• Peripancreatic collection possible
	• Abdominal pain, fever, and/or leucocytosis possible
	• Antibiotics and somatostatin analogues may be necessary
	• Delay in hospital discharge or readmission may be required
	Grade C:
	• Clinical stability maybe borderline
	• Treatment in an intensive care unit in many cases
	• Total parenteral/enteral nutrition
	• Intravenous antibiotics and somatostatin analogues necessary
	• Worrisome peripancreatic fluid collection that requires percutaneous drainage
	• Extended hospital stay
	• Often associated complications and postoperative mortality possible
▪ Delayed gastric emptying [13]	Delayed gastric emptying represents the inability to return to a standard diet by the end of the first postoperative week and includes prolonged nasogastric intubation of the patient. Three different grades (A, B, and C) were defined based on the impact of the clinical course and on postoperative management.

**Operation time**	From skin incision to closure of wound [min].

**Blood loss assessed by surgeons and anesthesists**	Intraoperative blood loss [ml].

**Hospital stay**	
▪ Initial postoperative hospital stay after randomization	Day of operation until day of discharge.
▪ Total hospital stay due to chronic pancreatitis within 24 months after randomization	Total amount of hospital days after randomization for any treatment due to chronic pancreatitis within 24 months.

**Reoperation due to recurrence of chronic pancreatitis**	Any surgical intervention for treatment of the pancreas at any time during the follow-up period.

**Weight gain**	Body weight [kg] assessed at all visits.

**New onset of diabetes mellitus requiring treatment**	Any continuous treatment (drugs) of diabetes lasting for 12 months.

**Development of exocrine insufficiency (continuous supplement of pancreatic enzymes necessary)**	Any continuous treatment (drugs) of exocrine insufficiency lasting for 12 months.

### Data management

All protocol-required information for the clinical data collected during the trial must be entered by the investigator, or by a designated representative, in the eCRF. The investigator is responsible for ensuring that all sections of the eCRF are completed correctly and that entries can be verified against source data. Any entry and correction in the Remote Data Entry System will be recorded automatically in an audit file. Once the documentation of a patient is completed and checked for plausibility the investigator is asked to date and sign it via electronic identification. Documentation of quality of life questionnaires will be done on paperbased CRFs.

Double data entry will be accomplished in order to ensure completeness, validity and plausibility of trial data.

### Safety evaluation and reporting of adverse events

Analysis of safety related data is performed with respect to frequency of serious adverse events, frequency of serious adverse events stratified by causality and frequency of morbidity in both treatment groups.

From the day the patient has signed informed consent until the regular end of trial at 24 months follow-up or until premature withdrawal of the patient, all serious adverse events will be documented on a "serious adverse event form" available in the investigator site file. A serious adverse event will be defined as an event, that results in death, is immediately life-threatening, requires or prolongs hospitalization or results in persistent or significant disability or incapacity. Serious adverse events will be classified to intensity (mild, moderate, severe), outcome (ongoing, recovered completely, recovered with sequelae, death, unknown) and causality (unrelated; possibly, probably or definitely related to trial intervention; not assessable). The assessment is based on surgical findings and the clinical course of the patient. It needs to be done by the investigators in the participating trial centres. They will be furnished with written recommendations on how to assess causality with trial intervention within the ChroPac trial.

Serious adverse events have to be reported by the attending physician to the principal investigator within 24 hours after the SAE becomes known.

### Statistical methods

#### Sample size calculation

The sample size calculation is based on the primary outcome and the primary analysis for the intention-to-treat population. The prior assumption based on the evaluation of 2 clinical trials is a mean intervention group difference of 10% for the EORTC QLQ-C30 scale "physical functioning" (range 0 to 100) with an estimated standard deviation of 20% 24 months after the surgical intervention [14,15].

With a two-sided level of significance α = 5% and a power of 1-β = 90%, a sample size of 86 patients per intervention group is required to detect this difference with a two-sided Student's t-test. It is assumed that an application of an analysis of covariance in the evaluation will lead to a reduction of the standard deviation. Furthermore, using the average of the EORTC QLQ-C30 scale "physical functioning" measured after 6,12 and 24 months instead of the value 24 months after the surgical intervention will presumably sharpen and stabilize the extent of the intervention group difference as also differences between the intervention groups that occur earlier after the surgery are taken into account. It can therefore be expected that this will increase the actual power of the trial as compared with the power calculated on the basis of the above-mentioned assumptions.

To compensate a potential loss in power caused by withdrawals, the sample size is increased by 15% and hence a total of 200 patients will be randomised.

#### Analysis

The confirmatory analysis is performed for the intention-to-treat (ITT) population.

The two-sided null-hypothesis for the primary outcome measure states that both surgical interventions lead to the same expected average QoL scores during 24 months after surgery. This null-hypothesis will be tested by application of an analysis of covariance that adjusts for age, centre and EORTC QLQ-C30 scale „physical functioning“ before surgery. Missing values will be replaced by multiple imputation [16].

Methods of descriptive data analysis will be used for the secondary outcome measures. Descriptive statistics will be calculated according to the scale level of the variables. Further analyses include the time course of the primary and secondary outcome measures. Additionally, sensitivity analyses will be conducted using different patient populations (per-protocol population excluding patients with relevant protocol violations), different imputation techniques for missing values, and different statistical methods for taking into account covariates. Graphical methods including scatter plots and boxplots will be used to visualize the findings of the trial.

The safety analysis includes calculation of frequencies and rates of complications and serious adverse events reported in the two intervention groups. All analyses will be done using SAS Version 9.1. or higher.

### Withdrawals

Patients are free to withdraw trial participation at their own request at any time and without giving reasons for their decision. Moreover, the primary investigator can withdraw study patients, if continuation of the trial would be detrimental to the patient's well being.

Withdrawals will be documented in the CRF and in the patient's medical records and all ongoing serious adverse events have to be followed up.

### Data Safety Monitoring Board (DSMB)

In case of any irregularities for example concerning the frequency or type of serious adverse events reported the principal investigator will inform the members of the independent DSMB without delay. At least once every 12 months, the DSMB will receive a written safety report. The result of the risk/benefit assessment will be reported to the principal investigator including recommendations concerning the continuation of the trial (Members of the DSMB: Gabriele Ihorst, PhD, Centre for Clinical Trials, University of Freiburg, Germany; Pierluigi Di Sebastiano, MD, Director General Surgery, San Giovanni Rotondo, Foggia, Italy; Helmut Witzigmann, Director Surgical Department Friedrichstadt Hospital, Dresden, Germany).

### Stopping guidelines

No interim analysis is planned for ChroPac. The trial can be prematurely closed by the coordinating investigator in consultation with the steering committee, the Data Safety Monitoring Board and the responsible biometrician for the following reasons:

• The incidence or severity of serious adverse events/morbidity in this trial indicates a potential health hazard caused by the trial treatment.

• It appears that patients' enrolment is unsatisfactory with respect to quality and/or quantity or data recording is severely inaccurate and/or incomplete.

• External evidence demanding a termination of the trial.

In case of premature closure, the ethics committee has to be informed.

### Trial organization and administration

#### Funding

ChroPac is funded by the DFG (Deutsche Forschungsgemeinschaft/project funding reference number SE 1682/2-1).

#### Monitoring

Clinical Monitoring will be performed by an independent institution already experienced due to the tasks in some other surgical trials (Coordinating Centre for Clinical Trials, Heidelberg, Germany) [17]. Monitoring procedures will be adapted to the trials-specific risk for the patients, interpretation of ICH-GCP (E6) and standard operating procedures to ensure patients' safety and integrity of the clinical data, e.g. primary outcome measure in adherence to study protocol. Prior to the start of the trial all participating centres will be personally trained and introduced into all trial-specific procedures during individual on site initiation visits. Regular on-site monitoring visits are planned at all sites depending on the recruitment rate and quality of the data (approximately one visit per site and year). The investigator must allow the monitor to look at all essential documents and must provide support at all times to the monitor. For 10% of all participants a 100% clinical source data verification for all clinical items is planned. In addition, all procedures will be predefined in a trial-specific monitoring manual. Queries of the data management (e.g. in case of missing values, implausibility etc.) have to be answered by the investigators contemporary to avoid that errors in data capture or entry will be carried forward.

### Ethical considerations

#### Approval

Before the start of the trial, the trial protocol, informed consent document and any other trial documents were submitted to the independent ethics committee on January 30^th ^2009. Ethics approval was reviewed on April 20^th ^2009 and finally issued on May 19^th ^2009.

#### Good Clinical Practice

The procedures set out in this trial protocol, pertaining to the conduct, evaluation and documentation of this trial, are designed to ensure that all persons involved in the trial abide by Good Clinical Practice and the ethical principles described in the current revision of the Declaration of Helsinki. The trial will be carried out in keeping with local legal and regulatory requirements.

#### Registration

The trial protocol was registered http://www.controlled-trials.com and was given a unique number for a world-wide identification of this trial (ISRCTN38973832).

### Translational research

The basic and clinical research aims to identify parameters which influence the clinical outcome and may be useful for prognostic or therapeutical decision making in future with main focus on differences between the two surgical techniques (DPPHR vs. PPW). Serum parameters (20 ml blood) are investigated and pancreatic tissue is analysed by histology, as well as biochemical and molecular biological techniques in a standardised fashion. The molecular alterations are analysed in detail and assessed for its clinical implications. Important parameters of pancreatic tissue in chronic pancreatitis include inflammation and fibrosis and the correlation to pain and pain relief: standardised histological reporting of the degree of inflammation and fibrosis, identification and quantification of expression of pancreatic enzyme activation (e.g. trypsin), cytokines, chemokines, growth factors (e.g. EGFR, TGF), stellate cells and its mediators, collagens are performed.

Since pain is the most frequent indication for pancreatic surgery, a main focus is the investigation of pancreatic and peripancreatic nerves, neurotransmitter, nerve growth factors, and the correlation of pain, chronic inflammation and its relief by pancreatic resection. Potential markers include substance P, NK-1R, CGRP, VIP, NPY, PACAP, bradykinine, eicosanoids. Diabetes mellitus (endocrine insufficiency) has major impact on the long-term outcome of patients with chronic pancreatitis. The correlation of endocrine insufficiency and reduction of endocrine cells and products (insulin, glucagons) in chronic pancreatitis are investigated. Chronic pancreatitis is a known risk factor for pancreatic cancer and premalignant lesions can be detected in inflammatory disease. Serum and tissue markers of carcinogenesis including K-ras, p-53 will be investigated and correlated with histology, clinical outcome, and surgical techniques used. All investigations will be performed centralised and all data stored in a separate database. This will be linked to the clinical database to answer the above-mentioned questions.

## Discussion

Surgical treatment of chronic pancreatitis is indicated in the case of pancreatic head enlargement with local complications (eg, stenosis of the common bile duct and/or main pancreatic duct, duodenal obstruction, compression of retropancreatic vessels), suspicion of malignancy, and most commonly intractable pain [3,4]. Even without inflammatory mass of the pancreatic head, a recent RCT demonstrated surgical treatment of being superior as compared with endoscopic drainage in patients with chronic pancreatitis and a distal pancreatic duct obstruction [10].

The preferred surgical treatment of chronic pancreatitis has been a focus of studies and debate for decades. Several modifications of DPPHR were developed in the last years in order to reduce surgical trauma and compared with PD and PPW in four single centre RCTs for safety and efficacy. The results have been pooled in a systematic review and meta-analysis demonstrating a superiority of DPPHR in terms of QoL, exocrine insufficiency, length of hospital stay, weight gain and occupational rehabilitation [11].

**Figure 3:**
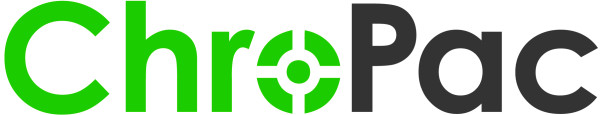
**ChroPac logo**.

Although DPPHR is technically demanding these techniques seem to be promising due to lesser operative trauma and the gastrointestinal and metabolic integrity by preservation of the duodenum [9]. Therefore, the role of DPPHR versus PD has to be evaluated now in a large-scale pragmatic randomised trial to demonstrate the effectiveness of DPPHR. Consequently, ChroPac was designed to focus on both the surgeons and patients perspectives in the comparison of the two main strategies (DPPHR vs. PD) (Figure 3).

### Strength and limitations

The research question of ChroPac is based on sufficient pilot data and since further single centre studies would not be able to strength the external validity, only a multicentre trial can demonstrate the superiority of DPPHR compared with PD in a setting close to daily practice. The Study Centre of the German Surgical Society (SDGC) as the coordinating organisation is experienced and has a proven track record in performing multicentre surgical trials, which highlight the SDGC's central role in trial design and administration and only go to strengthen the feasibility of the proposed trial [18]. An investigators meeting, which was conducted prior to the randomisation of the first patient represents strength of the ChroPac trial: 15 participants of the recruiting departments met at the SDGC in Heidelberg, Germany for a two day educational and practical workshop. While focussing on the explanation of the trials' rationale, surgical procedures, ethics, data management and monitoring within ChroPac, all participants attended surgical procedures for chronic pancreatitis on the second day at the operation theatres at the Department of General, Visceral and Transplantation Surgery, University of Heidelberg, Germany. Overall, the theoretical as well as the hands-on surgical training was highly appreciated and well-rated by the participants (range 1.3 - 2.6 with possible grades from 1 to 6). Moreover, there was general agreement regarding the rationale of the trial, primary outcome measure and feasibility of the trial (range 1.5 - 1.9 with possible grades from 1 to 4). As a result content, comprehensiveness and learning effect was rated to be excellent/very good (range 1.5 - 1.6 with possible grades from 1 to 6). Consequently, ChroPac promises optimal recruitment of study patients with good data quality by a both expertise and conform trial group.

However, the design of ChroPac raised some discussion during the design phase:

Firstly, the choice of the primary efficacy outcome measure *"quality of life" *was debated amongst surgeons, clinical investigators and methodological specialists. As a consequence, QoL will be evaluated by the EORTC QLQ-C30 subscore "physical functioning", which was validated and used in prior RCTs. Besides pain, several other aspects are summarized in this score and assessed from the patients' point of view, which justifies the validity of our primary outcome measure. As a secondary outcome all data of the full EORTC QLQ questionnaire will be collected in order to assess all dimensions of impairments and QoL.

Secondly, the specific trial design was a matter of debate. Since RCTs can be performed as efficacy (or explanatory) as well as effectiveness (or pragmatic) trials, the question to focus on internal validity (generation of the allocation sequence; allocation concealment; blinding; intention to treat analysis; complete outcome reporting and follow-up etc.) or on external validity (generalisability) was of major importance. Thus, performing an efficacy trial promises distinct evaluation of two surgical procedures rather than surgical strategies. ChroPac as an efficacy trial would have required explicitly defined surgical procedures (e.g. Frey versus PPW) and study patients. However, patients' compliance and generalisability is known to be low in these study designs and four single RCTs are already available [19,20]. As a result of the discussions during the investigator meeting the participants agreed and support the pragmatic approach of ChroPac:

Firstly, the available systematic review showed that the comparison of duodenum-preserving techniques versus pancreatoduodenectomy is currently hampered by the methodological weakness of only small available RCTs. Secondly, focussing on a sufficient sample size and optimal data quality the pragmatic approach with compact trial data acquisition and reduced study visits accounts for the compliance of both the investigators and the participating study patients. Thirdly, even a pragmatic approach allows a translational research part for further investigations of nature and mechanisms of chronic pancreatitis. Moreover, ChroPac evaluates a complex intervention and has to consider the expertise of different surgeons, peri-operative treatment regimens and concomitant interventions. Since these varying components reflect the current state-of-the-art treatment of chronic pancreatitis in centres of excellence for pancreatic surgery, ChroPac provides the most efficient evaluation of the present standard.

In summary, the ChroPac-Trial was designed to identify the best surgical strategy for treatment of chronic pancreatitis-either duodenum-preserving head resection or pancreatoduodenectomy. Focussing on a comparison of a general surgical strategy implies that the ChroPac-Trial will not be able to identify the best specific surgical technique in terms of available modifications of either PD or DPPHR. The aim of the ChroPac-Trial was therefore to maximize external validity while sustaining acceptable internal validity, which was accomplished by its pragmatic approach [21].

## Competing interests

The authors declare that they have no competing interests.

## Authors' contributions

MKD, TB, MWB and CMS designed and planned the ChroPac Trial. PC, CH, MG, HJS, JM and JW gave scientific input for the trials background, rationale and conduct. MK supervised the statistical background of the ChroPac Trial. JW conceived the translational research part. All authors read and approved the final manuscript.
